# Clinical characteristics and predictive factors of survival of 761 cancer patients on home parenteral nutrition: A prospective, cohort study

**DOI:** 10.1002/cam4.3064

**Published:** 2020-05-15

**Authors:** Paolo Cotogni, Taira Monge, Roberto Passera, Laura Brossa, Antonella De Francesco

**Affiliations:** ^1^ Unit of Parenteral Nutrition in Oncology Department of Internal Medicine Molinette Hospital Turin Italy; ^2^ Clinical Nutrition Department of Internal Medicine Molinette Hospital Turin Italy; ^3^ Nuclear Medicine Division Department of Radiology Molinette Hospital Turin Italy

**Keywords:** cancer survival, cohort study, home care, medical nutrition therapy, oncologic treatment, supportive care

## Abstract

**Background:**

Robust data reporting the survival of cancer patients on home parenteral nutrition (HPN) are lacking. The aim of this prospective, cohort study was to investigate clinical characteristics, predictive factors, and overall survival (OS) of adult‐malnourished cancer patients eligible for HPN according to the European guideline recommendations.

**Methods:**

During the study period, 1658 cancer patients were consecutively evaluated in a tertiary university hospital. Of these, 761 who received HPN were grouped into four cohorts according to the provision of supplemental PN (SPN) or total (TPN) and whether they received chemotherapy (CT^+^ or CT^‐^): SPN/CT^+^ (n = 376), TPN/CT^+^ (n = 99), SPN/CT^‐^ (n = 191), and TPN/CT^‐^ (n = 95). Patient demographics, nutritional status, cancer‐related characteristics, and prognostic scores assessed at HPN start. The primary outcome was OS.

**Results:**

Median OS was 8.9, 4.3, 5.7, and 2.2 months for the SPN/CT^+^, TPN/CT^+^, SPN/CT^‐^, and TPN/CT^‐^ cohorts, respectively. In multivariable analysis, predictors showing significant association with decreased survival were patient cohorts, modified Glasgow Prognostic Score (1 and 2 scores), weight loss (>15%) in the 3 months before HPN start, and TNM IV stage while protective factors of survival were Karnofsky Performance Status (>50), albumin level (>3.5 g/dL), oral protein intake, BMI (>20.5), and weight at HPN start.

**Conclusion:**

For the first time, in four different cohorts of cancer patients on HPN, clinical characteristics and survival were compared. This large study showed that survival is significantly correlated with patient characteristics at HPN start and that the presence of favorable factors may determine even a fourfold increase in survival. These data are expected to assist physicians in the appropriate prescription of HPN.

## Graphical abstract

This is the largest, prospective, clinical study investigating survival exclusively of adult malnourished cancer patients receiving home parenteral nutrition (HPN). This study showed that survival is significantly correlated with patient characteristics at HPN start and that the presence of favorable factors may determine even a 4‐fold increase in survival.

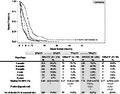

## 1 INTRODUCTION

In cancer patients, an impaired nutritional status impacts negatively on functional status, quality of life, tolerance to oncologic treatments, rates of hospitalization, length of stay, and survival.^1^ International guidelines recommend to regularly screen all cancer patients for nutrition impact symptoms and clinical signs of malnutrition and, if found at risk, to design personalized nutritional interventions.^2^, ^3^ Nutritional support in oncologic patients is a step‐by‐step intervention,^1^ starting from dietary counseling and oral nutritional supplements (ONS) to Medical Nutrition therapy (MNT), either enteral nutrition (EN) or parenteral nutrition (PN).^4^, ^5^ In case that oral nutrition remains inadequate despite counseling and ONS and EN are not feasible, insufficient, or contraindicated, home PN (HPN) ensures that patients receive adequate nutritional therapy.^6^ This is the case in patients with chronic severe enteral food intolerance (untreatable nausea, vomiting, abdominal pain, malabsorption, or diarrhea) or with severe intestinal insufficiency due to malignant inoperable bowel obstruction (peritoneal carcinomatosis, intra‐abdominal recurrences), short bowel syndrome, radiation enteritis, and high‐output ileostomy or fistulas.^1^, ^7^ HPN can be total (TPN) when patients have no or negligible ONS/EN nutrition (<200 kcal/day)^8^ or supplemental PN (SPN). Generally, SPN at home provides 1000‐1250 kcal per day, from three to six times per week in patients with residual—but insufficient—oral food intake.

Prevalence of HPN in cancer patients throughout the world reflects differing practices, with this variability possibly attributable to different reimbursement policies and economic resources allocation as well as to cultural, ethical, and social aspects among countries.^9^, ^10^, ^11^, ^12^, ^13^ Additionally, the low level of the available evidence due to lack of randomized controlled trials (RCTs),^14^ together with the limited knowledge and diffusion of guidelines on nutrition in cancer patients,^15^, ^16^, ^17^ may contribute as well to this heterogeneity. In 2005, a survey to determine the prevalence of home MNT in Italy reported that in adults the greatest prevalence of HPN was observed in oncologic patients; in particular, a positive association was found between the number of years since the regulation was issued and home MNT prevalence.^18^ Since 1986, in the Piedmont Region both home EN and PN have been regulated by specific laws that stated the need of consistent screening of cancer patient nutritional status and their eligibility for HPN is assessed according to specific clinical practice guidelines. For years our Unit has been collecting data with the goal to determine factors affecting clinical practice, complications, and outcomes of cancer patients receiving HPN, in collaboration with the Regional Health Council, oncology units, and general practitioners.^19^, ^20^, ^21^, ^22^, ^23^, ^24^, ^25^

HPN cannot be studied in a randomized design, due to the fact that the control arm would be no feeding and that would be considered unethical in persons with severely compromised food intake or intestinal failure. Therefore, alternative study designs are required to interpret the potential benefit of this form of nutritional therapy.

The aim of this prospectively conducted study was to increase the knowledge of real‐world data on the use of HPN in cancer patients reporting a 7‐year experience of our Unit. In particular, we provided a detailed description of the pathway leading to the delivery of HPN in oncologic patients accordingly to daily clinical practice, clinical characteristics, predictive factors of survival at the time of HPN start, and survival since HPN start.

## 2 METHODS

### 2.1 Study design

This was a prospective, cohort study conducted in a 1200‐bed tertiary university hospital. From 1 June 2008 through 31 May 2015, all consecutive adult cancer patients who were candidates for HPN were eligible for enrollment. Study methods were conducted and reported in accordance with the Strengthening the Reporting of Observational Studies in Epidemiology (STROBE) reporting guideline for cohort studies.

Our criteria for accepting patients in the HPN program followed the European guideline recommendations for eligibility^5^ and included the following: proven and prolonged failure to meet nutritional requirements by oral or enteral route (no food for more than 1 week or less than 60% of requirement for more than 1‐2 weeks), with a potential risk of earlier death due to malnutrition rather than from cancer progression; life expectancy > 2 months; Karnofsky Performance Status (KPS) ≥50; adequate control of pain and other severe symptoms (dyspnea, vomiting); absence of severe organ dysfunctions; written informed consent confirming that the patient accepted this modality of nutritional support; having a central venous catheter (CVC); approval by the physician responsible for HPN, the oncologist and the general practitioner; presence of environmental conditions compatible with HPN; availability of an in‐home caregiver; and availability of a specifically trained nursing team dedicated to the patient home care, as provided by the Public Health Service.^23^ Exclusion criteria for HPN were as follows: capability to meet the nutritional requirements by oral or enteral route; KPS < 50; uncontrolled symptoms; severe organ dysfunctions (heart, respiratory, liver, and renal); lack of an in‐home caregiver and HPN refusal by the patient.

The evaluation of eligibility of a cancer patient for the HPN program was requested to our Unit mainly by oncologists, as also surgeons, internal medicine physicians, and general practitioners. Inpatients were assessed during the consultations carried out in the wards before discharge, while ambulatory patients were assessed in a dedicated hospital outpatient department in the Comprehensive Cancer Center. All patients were assessed for eligibility by the physician (PC) and the dietician (TM) responsible for HPN. At baseline (at HPN start), data recorded included anthropometric (actual body weight, body mass index [BMI], weight loss in the last 3 months) and clinical‐oncological assessments (tumor site and stage, anticancer treatments), and assessment of residual oral feeding, if present. Performance status was graded using the WHO/Eastern Cooperative Oncology Group (ECOG) scale^26^ and the KPS.^27^ Systemic inflammation was estimated using serum C‐reactive protein (CRP) and albumin, and inflammatory response was graded according to the modified Glasgow Prognostic Score (mGPS; 0 = CRP ≤10 mg/L + any albumin; 1 = CRP >10 mg/L + albumin ≥3.5 g/dL; 2 = CRP >10 mg/L + albumin <3.5 g/dL).^28^ Nutritional status was assessed using the Patient‐Generated Subjective Global Assessment (PG‐SGA), which combines qualitative and semi‐quantitative data to yield a comprehensive malnutrition score (A = well‐nourished; B = suspected malnutrition or moderate malnutrition; or C = severely malnourished.^29^ Three days was the mean time between eligibility for HPN and PN start at home.

After HPN start, all patients were closely monitored by the physician responsible for HPN (PC) through regularly scheduled and structured telephone interviews (at least every 15 days) and home visits by the nursing team and general practitioner (initially daily for 2‐3 weeks, and at least every 7 days thereafter). After adequate training, home caregivers administered HPN. Telephone assistance was available for patients as well as their caregivers and health‐care providers at all times. HPN was delivered to 98.4% of patients using standard commercially manufactured ready‐to‐use bags containing amino acids, electrolytes, glucose, and lipids, overnight for 10‐14 hours per day through a CVC. HPN regimen was individually designed to meet protein, calorie, and fluid requirements; generally, HPN was prescribed to provide 25‐30 kcal/kg/day, depending on the patient activity of daily living, and an amino acid supply of 1‐1.5 g/kg/day. Every 30 days from HPN start (± 5 days), an outpatient re‐evaluation by both the physician and the dietitian (including a 24‐hour food recall) was performed.

Eligible patients without an in‐home caregiver were admitted to hospice facilities, while patients who refused HPN, but with an in‐home caregiver, were assisted at home by palliative care teams (nurse and physician). All these patients received artificial hydration (balanced salt solutions) through a CVC according to their needs.

All patients were followed up until withdrawal of HPN or death. HPN was withdrawn in case of worsening clinical state (onset of severe organ dysfunction or uncontrolled symptoms; downgrading of performance status; estimated life expectancy of hours to days and patient will).^30^ Overall survival (OS) was calculated as the number of days between the date of HPN start and the date of patient death from any cause, with censoring at the date of last follow‐up assessment in alive subjects (on 28 February 2019).

Ethics committee approval was obtained and written informed consent was obtained from each patient prior to any procedures. The consent to participate was obtained from the chief investigator (PC). The latter submitted an update report monthly to the regional competent authority and annually or on request to the approving research ethics committee.

### 2.2 Statistical Analysis

All analyses were tabulated and a description of patient clinical characteristics was provided on the overall population as well as selected cohorts: patients were grouped into four mutually exclusive cohorts depending on type of HPN (SPN or TPN) and whether they received chemotherapy (CT^+^ or CT^‐^). Qualitative variables were described in terms of frequencies and percentages, while quantitative variables were reported as mean value and standard deviation (SD) or median and 95% confidence interval (CI). The primary outcome was OS measured in months between the date of HPN start and the date of death. Patients still alive at the end of the observation period were censored. Kaplan‐Meier curves plotting time to death since HPN start were also presented for the four cohorts with log‐rank tests. Study variables were fitted into a Cox proportional hazards model to estimated hazard ratios (HRs) and the associate 95% CI. A stepwise variable selection approach was used to enter only significant predictors into the final model (threshold 0.1). Results were considered significant if *p* value <0.05. All the analyses were performed using SAS software (Version 9.4).

## 3 RESULTS

### 3.1 Assessment of eligibility for HPN

**Figure 1:**
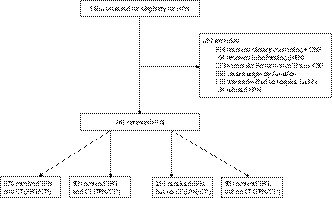
Study flow chart. HPN: home parenteral nutrition; ONS: oral nutritional supplements; HEN: home enteral nutrition; SPN: supplemental parenteral nutrition; TPN: total parenteral nutrition; CT+: chemotherapy received; CT‐: no chemotherapy received

During the study period, 1658 patients were consecutively evaluated. About 897 (54.1%) were excluded as they did not meet the inclusion criteria (Figure 1). In particular, 314 (18.9%) were judged to be eligible for a program providing dietary counseling and ONS; 64 (3.9%) were eligible for tube feeding (home EN); 213 (12.8%) had a KPS < 50; 153 (9.2%) patients presented with severe organ dysfunction. Other reasons for non‐administration were admission to hospice facility due to lack of an in‐home caregiver (119, 7.2%) or HPN refusal by the patient (34, 2.1%). Finally, 761 (45.9%) received HPN. Among patients who started HPN, 376 (49.4%) received SPN and CT (SPN/CT^+^); 99 (13.0%) received TPN and CT (TPN/CT^+^); 191 (25.1%) received SPN but not CT (SPN/CT^‐^); and 95 (12.5%) received TPN but not CT (TPN/CT^‐^).

The overall population required HPN mainly because the oral food intake was severely compromised by the presence of peritoneal carcinomatosis (30%) or intra‐abdominal recurrences (58%); less frequently (12%) because of short bowel syndrome, high‐output ileostomy or fistulas, and not feasible or tolerated EN.

The rate of catheter‐related bloodstream infections (CRBSIs) was 0.29 per 1000 catheter‐days while the rate of overall other catheter‐related complications (CRCs; local infection, mechanical, and venous thrombosis) was 0.79 per 1000 catheter‐days. Eight patients required hospitalization due to CRBSI and one of them died. No clinically relevant HPN‐related metabolic complications occurred. There were no significant differences in complications or adverse events among the four cohorts.

### 3.2 Patient characteristics

**Figure 2:**
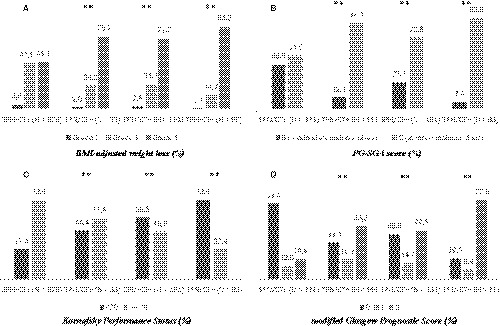
Differences in patient distribution (%) for the four groups identified (by the type of parenteral nutrition and whether they received chemotherapy during the study period). Differences are according to (a) BMI‐adjusted weight loss categories, (b) Patient‐Generated Subjective Global Assessment (PG‐SGA) score, (c) Karnofsky Performance Status, and (d) modified Glasgow Prognostic Score. ** *P* < .01 (SPN/CT + was the reference group when compared with TPN/CT+; SPN/CT‐; TPN/CT‐).SPN: supplemental parenteral nutrition; TPN: total parenteral nutrition; CT+: chemotherapy received; CT‐: no chemotherapy received; BMI: body mass index

In Table 1, the characteristics of patients at HPN start. Regarding age, patients who did not receive chemotherapy (CT^‐^) were older compared with those in the SPN/CT^+^ group, with more patients in the ≥ 70 years category. The cohorts of patients who did not receive chemotherapy presented a proportion of underweight patients of 50% or above. In particular, the SPN/CT^‐^ group had lower weight and BMI compared with SPN/CT^+^ (Table 1). Patient distribution in weight loss and BMI‐adjusted weight loss categories (Figure 2A) was different between SPN/CT^+^ and the other cohorts, suggesting that patients in the SPN/CT^+^ group are less likely to be in the highest categories of weight loss (> 15%), even when adjusted for BMI (BMI‐adjusted weight loss grade 4). Similarly, severely malnourished patients (PG‐SGA rating of C) were more present in the TPN/CT^+^, SPN/CT^‐^, TPN/CT^‐^ groups when compared with SPN/CT^+^ (Figure 2B). When we looked at residual oral food intake at the start of HPN, the number of patients reporting better oral calorie and protein intake was higher in the SPN/CT^+^ group compared with SPN/CT^‐^ (Table 1).

**Table 1:** Patient characteristics by type of parenteral nutrition and whether they received chemotherapy during the study period

	Overall (N = 761)		Chemotherapy received							No chemotherapy received					
			SPN/CT^+^ (N = 376)			TPN/CT^+^ (N = 99)				SPN/CT^‐^ (N = 191)			TPN/CT^‐^ (N = 95)		
	N	%	N	%	N		%	*P^1^*	N		%	*P^2^*	N	%	*P^3^*
Sex															
Female	380	49.9	197	52.4	41		41.4		102		53.4		40	42.1	
Age (years)															
Mean (SD)	62.90	(10.28)	60.07	(9.15)	62.07		(9.89)		66.12		(10.84)	*<.01*	68.49	(9.73)	*<.01*
Age categories (years)															
<70	555	72.9	325	86.4	75		75.8		113		59.2		42	44.2	
≥70	206	27.1	51	13.6	24		24.2	*<.01*	78		40.8	*<.01*	53	55.8	*<.01*
Actual weight (kg)															
Mean (SD)	58.42	(10.92)	59.56	(11.32)	58.74		(10.12)		56.11		(10.23)	*<.01*	58.21	(10.92)	
BMI (kg/m^2^)															
Mean (SD)	21.37	(3.76)	21.75	(4.01)	21.62		(3.60)		20.54		(3.12)	*<.01*	21.23	(3.84)	
BMI class															
<20.5 kg/m^2^	334	43.9	149	39.6	37		37.4		101		52.9		47	49.5	
≥ 20.5 kg/m^2^	427	56.1	227	60.4	62		62.6		90		47.1	*<.01*	48	50.5	
Weight loss (%) categories^a^															
≤10.0%	233	30.6	162	43.1	12		12.1		52		27.2		7	7.4	
10.1%‐15%	187	24.6	109	29.0	21		21.2		40		20.9		17	17.9	
15.1%‐20%	181	23.8	59	15.7	35		35.4		49		25.7		38	40.0	
>20%	160	21.0	46	12.2	31		31.3	*<.01*	50		26.2	*<.01*	33	34.7	*<.01*
BMI‐adjusted weight loss															
Grade 2	26	3.4	18	4.8	2		2.0		5		2.6		1	1.1	
Grade 3	264	34.7	177	47.1	24		24.2		49		25.7		14	14.7	
Grade 4	471	61.9	181	48.1	73		73.7	*<.01*	137		71.7	*<.01*	80	84.2	*<.01*
PG‐SGA															
B^b^	242	31.8	171	45.5	12		12.1		52		27.2		7	7.4	
C^c^	519	68.2	205	54.5	87		87.9	*<.01*	139		72.8	*<.01*	88	92.6	*<.01*
Oral calorie intake (kcal/day)															
<500	322	42.3	65	17.3	—		—		63		33.0		—	—	
500‐1000	409	53.8	291	77.4	—		—		118		61.8		—	—	
>1000	30	3.9	20	5.3	—		—		10		5.2	*<.01*	—	—	
Oral protein intake (g/day)															
<20	353	46.39	84	22.3	—		—		75		39.3		—	—	
≥20	408	53.61	292	77.7	—		—		116		60.7	*<.01*	—	—	
Tumor site															
Ovary	47	6.2	24	6.4	6		6.1		11		5.8		6	6.3	
Gastrointestinal	564	74.1	293	77.9	72		72.7		134		70.2		65	68.4	
Other	150	19.7	59	15.7	21		21.2		46		24.1		24	25.3	
Metastatic cancer															
No	301	39.6	174	46.3	27		27.3		67		35.1		33	34.7	
Yes	460	60.5	202	53.7	72		72.7	*<.01*	124		64.9	*<.05*	62	65.3	*<.05*
Peritoneal carcinomatosis															
No	533	70.0	288	76.6	54		54.6		126		66.0		65	68.4	
Yes	228	30.0	88	23.4	45		45.5	*<.01*	65		34.0	*<.01*	30	31.6	
Cancer staging															
II/III	220	28.9	137	36.4	19		19.2		47		24.6		17	17.9	
IV	541	71.1	239	63.6	80		80.8	*<.01*	144		75.4	*<.01*	78	82.1	*<.01*
KPS															
50	102	13.4	8	2.1	11		11.1		43		22.5		40	42.1	
60	222	29.2	95	25.3	33		33.3		65		34.0		29	30.5	
70	387	50.9	230	61.2	55		55.6		77		40.3		25	26.3	
80‐90	50	6.6	43	11.4	.		.	*<.01*	6		3.1	*<.01*	1	1.1	*<.01*
Albumin															
<3.5 g/dL	430	56.5	163	43.4	62		62.6		123		64.4		82	86.3	
≥3.5 g/dL	331	43.5	213	56.7	37		37.4	*<.01*	68		35.6	*<.01*	13	13.7	*<.01*
CRP															
≤10 mg/L	390	51.2	261	69.4	33		33.3		78		40.8		18	18.9	
>10 mg/L	371	48.8	115	30.6	66		66.7	*<.01*	113		59.2	*<.01*	77	81.1	*<.01*
mGPS															
0	390	51.3	261	69.4	33		33.3		78		40.8		18	19.0	
1	99	13.0	45	12.0	18		18.2		28		14.7		8	8.4	
2	272	35.7	70	18.6	48		48.5	<.01	85		44.5	<.01	69	72.6	<.01

All patient characteristics were at the time of home parenteral nutrition start.

Abbreviations: BMI, body mass index; CRP, C‐reactive protein; CT^−^, no chemotherapy received; CT+, chemotherapy received; ECOG, Eastern Cooperative Oncology Group; KPS, Karnofsky Performance Status; mGPS, modified Glasgow Prognostic Score; PG‐SGA, Patient‐Generated Subjective Global Assessment; SD, standard deviation; SPN, supplemental parenteral nutrition; TPN, total parenteral nutrition.

*P* ^1^: the groups compared were SPN/CT^+^ vs. TPN/CT^+^; *P* ^2^: SPN/CT^+^ vs. SPN/CT^‐^; *P* ^3^: SPN/CT^+^ vs. TPN/CT^‐^. Where the comparison was significant, *P*‐value is reported.

^a^ In the last 3 months before home parenteral nutrition (HPN) start.

^b^ Moderately malnourished or suspected malnutrition.

^c^ Severely malnourished.

Overall, most frequent tumors observed among patients starting HPN were those involving the digestive system. In particular, stomach cancer was the most frequently observed tumor for all cohorts (36%, 37%, 20%, and 24% for SPN/CT^+^, TPN/CT^+^, SPN/CT^‐^, and TPN/CT^‐^, respectively), followed by pancreatic and colon cancers. The number of patients with metastatic cancer, peritoneal carcinomatosis, and cancer stage IV was lower in the SPN/CT^+^ compared with the other three cohorts (Table 1). A similar difference in patient distribution was observed for the KPS (measured in four categories, Table 1, or as binary variable, Figure 2C) and the mGPS (Figure 2D). Patients who did not undergo chemotherapy and received TPN (TPN/CT^‐^) had a lower KPS (73% had KPS 50 or 60), lower albumin levels (86% had an albumin value < 3.5 g/dL), higher CRP levels (81% had a CRP value > 10 mg/L), and a worse mGPS (73% had an mGPS of 2). In contrast, patients who underwent chemotherapy and received SPN (SPN/CT^+^) had a higher KPS, higher albumin levels, lower CRP levels, and a better mGPS (Table 1).

### 3.3 Patient survival

**Figure 3:**
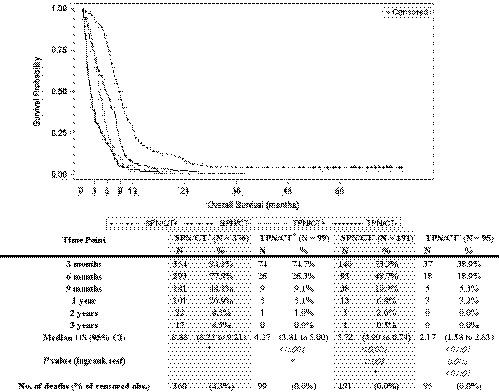
Overall survival. Overall survival (in months) since home parenteral nutrition start reported as Kaplan‐Meier curves that identify the different cohorts; percent of patients alive at different time‐points; median survival and 95% CI. * Reference group for the log‐rank test. SPN: supplemental parenteral nutrition; TPN: total parenteral nutrition; CT+: chemotherapy received; CT‐: no chemotherapy received; CI: confidence interval

Maximum follow‐up time was just over 6 years and no patients were lost to follow‐up. In Figure 3, Kaplan‐Meier curves for survival since HPN start in the different cohorts of treatment show a sharp decline in the first 12 months. The proportion of patients who were alive after the first 6 months of follow‐up since HPN start went from 77.9% for the SPN/CT^+^ group to 26.3%, 48.7%, and 18.9% (TPN/CT^+^, SPN/CT^‐^, and TPN/CT^‐^, respectively). Median OS in the whole population was 6.7 months (95% CI, 6.4 to 7.1), but was significantly different in the four cohorts analyzed: patients in the SPN/CT^+^ group survived significantly longer than those in the other cohorts (Figure 2). When comparing the other three cohorts between them, median OS differences were statistically significant in log‐rank test analysis (Table of Figure 3).

All variables were analyzed to identify independent predictors of survival in univariate and multivariable analysis with the exception of peritoneal carcinomatosis that was excluded in the final model due to collinearity (Table 2). Differences in survival between SPN/CT^+^ and the other cohorts were confirmed in univariate and fully adjusted analysis with the TPN/CT^‐^ group having the highest HR. Similarly, the protective factors of survival at HPN start were as follows: a higher KPS (>50), higher oral protein intake (>20 g/day), normal albumin levels (≥3.5 g/dL), BMI (>20.5), and weight, while the predictors that decreased survival at HPN start were as follows: mGPS of 1 or 2, weight loss in the last 3 months (>15%), and being on cancer stage IV compared with II/III. Conversely, age (≥70), weight 3 months before HPN start, decreased oral calorie intake, BMI‐adjusted weight loss grade 4, PG‐SGA score rating of C, ECOG scale of 2, and CRP > 10 mg/L were significant only in univariate analysis.

**Table 2:** Univariate analysis and Cox's proportional hazards models to assess the effect on patient survival of the variables considered in the study

	Univariate			Stepwise variables selection		
	HR	95% CI	*P*	HR	95% CI	*P*
Cohort subgroups						
SPN/CT^+^ (N = 376)	1			1		
TPN/CT^+^ (N = 99)	2.99	2.38 to 3.76	*<.001*	1.35	1.03 to 1.77	*.028*
SPN/CT^‐^ (N = 191)	2.01	1.68 to 2.40	*<.001*	1.46	1.20 to 1.77	*<.001*
TPN/CT^‐^ (N = 95)	4.57	3.62 to 5.77	*<.001*	1.71	1.28 to 2.29	*<.001*
**Sex**						
Male	1			1		
Female	0.89	0.77 to 1.03	*.117*	0.64	0.54 to 0.76	*<.001*
Age categories (years)						
<70	1					
≥70	1.77	1.50 to 2.08	*<.001*			
**Actual weight (kg, continuous)**	0.98	0.98 to 0.99	*<.001*	0.97	0.96 to 0.98	*<.001*
BMI class						
<20.5 kg/m^a^	1			1		
≥20.5 kg/m^2^	0.71	0.61 to 0.82	*<.001*	1.25	1.02 to 1.54	*.033*
Weight loss^a^ (%) categories						
≤10.0%	1			1		
10.1%‐15%	1.56	1.28 to 1.90	*<.001*	1.11	0.89 to 1.37	*.355*
15.1%‐20%	6.91	5.53 to 8.65	*<.001*	2.45	1.87 to 3.21	*<.001*
>20%	9.42	7.45 to 11.91	*<.001*	2.94	2.22 to 3.90	*<.001*
Oral calorie intake (kcal/day)						
>1000	1					
500‐1000	1.86	1.24 to 2.80	*.003*			
<500	4.97	3.28 to 7.52	*<.001*			
Oral protein intake (g/day)						
<20	1			1		
≥20	0.37	0.32 to 0.43	*<.001*	0.71	0.58 to 0.86	*<.001*
Tumor site						
Ovary	1					
Gastrointestinal	0.88	0.65 to 1.18	*.392*			
Other	1.18	0.85 to 1.63	*.336*			
Cancer staging						
II/III	1			1		
IV	2.80	2.36 to 3.33	*<.001*	1.34	1.01 to 1.78	*.044*
KPS						
50	1			1		
60	0.58	0.46 to 0.74	*<.001*	0.69	0.53 to 0.89	*.005*
70	0.23	0.18 to 0.29	*<.001*	0.63	0.49 to 0.82	*<.001*
80‐90	0.08	0.06 to 0.12	*<.001*	0.33	0.22 to 0.50	*<.001*
ECOG scale						
1	1					
2	3.35	2.87 to 3.92	*<.001*			
Albumin						
<3.5 g/dL	1			1		
≥3.5 g/dL	0.37	0.32 to 0.43	*<.001*	0.69	0.55 to 0.87	*.001*
mGPS						
0	1			1		
1	3.63	2.87 to 4.57	*<.001*	3.17	2.39 to 4.22	*<.001*
2	19.69	15.74 to 24.63	*<.001*	8.35	6.35 to 10.97	*<.001*

All patient characteristics were at the time of home parenteral nutrition start.

Abbreviations: BMI, body mass index; CI, confidence interval; CT^‐^, no chemotherapy received; CT+, chemotherapy received; ECOG, Eastern Cooperative Oncology Group; KPS, Karnofsky Performance Status; mGPS, modified Glasgow Prognostic Score; SPN, supplemental parenteral nutrition; TPN, total parenteral nutrition.

^a^ In the last 3 months before home parenteral nutrition (HPN) start.

## 4 DISCUSSION

In the last 20 years, survival in cancer outpatients on PN has been investigated in a number of observational studies.^8^, ^11^, ^31^, ^32^, ^33^, ^34^, ^35^, ^36^, ^37^, ^38^ Some studies reported that a survival longer than expected in aphagic or severely hypophagic cancer patients can be achieved through PN.^8^, ^11^, ^32^ This especially occurred in patients presenting favorable prognostic factors (KPS, mGPS, and tumor stage)^8^, ^31^, ^34^, ^35^, ^36^ or responding to chemotherapy.^33^ In these studies, median OS varied widely: 45 days,^33^, ^35^ 3‐4 months,^8^, ^34^, ^37^, ^38^ or 5‐6 months.^11^, ^31^, ^32^, ^36^

However, all these papers have one or more of the following limitations: patient inclusion criteria did not comply with guideline recommendations for PN administration (KPS < 50)^8^, ^31^, ^34^, ^35^, ^36^ or short life expectancy^33^, ^35^; a small sample size of 50‐150 patients^11^, ^31^, ^32^, ^33^, ^34^, ^35^, ^37^ or less^36^, ^38^; design of the study (retrospective instead of prospective)^32^, ^34^, ^35^, ^38^; inclusion of patients with both cancer and noncancer diseases^37^, ^38^; inclusion of patients with different oncologic approaches (treated and nontreated patients)^11^, ^33^, ^36^, ^38^; and different setting of PN administration (hospital or hospice instead of home).^32^, ^33^ Therefore, it has not been possible to highlight a possible survival advantage induced by HPN so far.

To our knowledge, this is the largest, prospective, clinical study investigating survival exclusively of adult‐malnourished cancer patients receiving PN at home. Moreover, the originality of our study is that we investigated the OS of four cohorts of cancer patients on HPN grouped according to the provision of SPN or TPN and whether they received chemotherapy. Despite the differences in median OS reported between cohorts, the aim of this study was not to draw inference on the benefit of the type of PN (SPN or TPN) or chemotherapy, given the differences between the four cohorts at HPN start. For instance, the longer median OS in the SPN/CT^+^ group was not surprising as patients in this cohort were younger, suffered less weight loss and cancer progression in the months leading to HPN start, had better nutritional status and prognostic scores (KPS and mGPS). Therefore, instead of focusing on comparison, results from this observational study should be considered as a detailed description of each cohort.

Interestingly, the cohorts of patients who showed a higher median survival (SPN groups) are often considered the ones less likely to receive HPN because of the presence of residual oral food intake.^17^, ^39^, ^40^ Given the median OS of nearly 9 months, this study adds to the discussion around the benefit of SPN in advanced cancer patients receiving chemotherapy.^20^, ^41^, ^42^ Of course, no direct inference on the benefit of SPN can be drawn from this study given that no control group without PN was available.

Indeed, RCTs remain the gold standard for this comparison. Regarding survival outcome, two underpowered RCTs reported that PN was neither superior to fluid administration in patients with days or a few weeks of expected survival^43^ nor to dietary counseling in patients with oral energy intake above 75% of estimated needs and receiving chemotherapy.^44^ However, the major limitation of both RCTs was that patient inclusion criteria did not comply with guideline recommendations for PN administration. Actually, PN is neither indicated in patients with short life expectancy nor in patients with sufficient ONS or EN intake.^2^, ^45^

Indications that higher KPS scores increase the probability of survival have been reported in observational^8^, ^34^, ^35^ and survival prediction studies,^19^ although KPS scores were reported as lower or equal/higher than 50. Differently, in this study we included only patients with KPS ≥ 50 according to the guideline recommendations for eligibility for HPN. Consequently, given the large sample of patients, survival analyses in this study were able to highlight how even small increases in KPS score (10‐point increases) could significantly have a protective effect on survival.

The mGPS is an inflammation‐based prognostic score widely used in predicting survival in cancer patients.^28^, ^46^, ^47^ Specifically, when measured at HPN start, elevated mGPS scores have been associated with a higher risk of mortality^8^, ^34^ and has also been incorporated in a nomogram to predict survival in cancer patients on HPN.^19^ Our study confirmed the value of mGPS as a strong predictor of survival while PG‐SGA, ECOG scale, and BMI‐adjusted weight loss^48^ failed to provide prognostic contribution in the multivariable analysis. Similarly, we confirmed that tumor spread (TNM stage IV at HPN start) was a predictor of reduced survival in these patients.^19^

In multivariable analysis, other predictors of survival were higher oral protein intake and normal albumin levels that showed protective effect on mortality. The role of albumin as prognostic factor in patients with advanced cancer is known,^46^ although little indications that albumin levels could be a significant predictor in HPN patients have been reported.^31^, ^34^, ^35^

In this study, we also reported how body weight at HPN start was a significant factor and each kilogram of weight could potentially reduce the risk of death by 3%. Indeed, the suggestion that in cancer patients, especially those exposed to cancer therapy, a higher body weight could be beneficial to survival has already been proposed.^49^ However, a number of variables associated with body weight and weight loss are similar and therefore unlikely be significant in the multivariate analysis.

In our study, some patients died before the estimated life expectancy of 2 months; therefore these "estimates" are inadequate. Our survival analysis lends itself to the potential development of a prognostic index, which including all the key variables could be extremely powerful in predicting short survival probabilities (few weeks). Indeed, since June 2015 we adopted a nomogram to predict survival in cancer patients and replace the estimated life expectancy as inclusion criteria for HPN eligibility.^19^

Some physicians are concerned that initiating HPN may lead to onset of CRCs, especially in patients receiving chemotherapy. An important message for oncologists is that, as in our early experience,^22^, ^23^ we reported that HPN can be safely carried out (low rate of CRBSI and other CRCs) in cancer patients, even if receiving chemotherapy, consistent with the current practice of HPN.

However, HPN safety is mainly assured if the evaluation of eligibility for HPN program is performed by physicians and dieticians expert in nutrition in oncology as well as if patients and caregivers are adequately trained at home by a specialized nursing staff and are carefully monitored in the follow‐up.^50^ In this study, eligibility for HPN program was assessed according to a set of criteria following the European guidelines^4^, ^5^ that assured a regulated access to this nutritional intervention. As a result is noteworthy that the cohort of patients who received HPN represents only the 46% of the initial 1658 patients referred for eligibility for HPN to our Unit.

When compared with previous studies in this research field, our study has many strengths: [1] was a prospectively conducted study; [2] data were collected through a clinical observation carried out over a 7‐year period; [3] the study population consisted of 761 adult patients; [4] all patients were evaluated as eligible for HPN program according to the guideline recommendations; [5] only cancer patients were included; [6] all patients were malnourished; [7] only patients receiving PN at home were included; [8] two thirds of patients were receiving chemotherapy during the study period; [9] there were no missing data; and [10] no patients were lost at follow‐up.

In our Region, institution of HPN is the standard of care in these cancer patients, and furthermore it has been instituted by established criteria according to oncology nutrition clinical practice guidelines.^4^, ^5^ This gives us an opportunity to determine which factors may be associated with survival in patients fed intravenously. This study could not be done in other countries where, for cultural (not scientific) reasons, HPN is not that standard of care.

The main limitation of this study is the lack of randomization; however, an RCT is ethically unacceptable because a control group of patients who are aphagic or severely hypophagic and do not get any nutritional support is at risk of earlier death due to malnutrition rather than from cancer progression. Besides, this is a single‐center study; however, our results may be generalizable to other populations of cancer patients on HPN according to guideline recommendations. Finally, patients’ cancer treatment plans were not reported; however, a detailed description of the chemotherapy and radiotherapy regimens adopted in the different sites and stages of the tumors was beyond the aims of this paper.

## 5 CONCLUSION

With the increasing accessibility to effective anticancer treatments and their ability to transform the trajectory of the advanced cancer into a chronic condition, a growing number of patients are expected to be in need of HPN. However, there is a lack of robust data reporting the survival of these patients on HPN.

In summary, this prospective study in a large population of cancer patients on HPN showed that survival is significantly correlated with patient characteristics at HPN start and that the presence of favorable factors may determine even a fourfold increase in survival. These data reporting real‐world data on the use of HPN are expected to assist physicians in appropriate prescription of HPN, patients and their home caregivers to make better‐informed treatment decisions, and health‐care professionals and institutions to develop best practices for HPN.

## CONFLICT OF INTEREST

PC reported honoraria for speaking and teaching from Baxter. The other authors declare that they have no competing interests.

## AUTHOR CONTRIBUTIONS

PC was the chief investigator, designed the study and developed the protocol. PC, TM, and LB participated in recruitment of patients. PC and RP developed and carried out the statistical analysis plan. PC, TM, and ADF coordinated the data collection and regulatory and governance requirements. PC and RP interpreted the data. PC wrote the draft manuscript. All authors contributed to the review and amendments of the manuscript for important intellectual content and approved this final version for submission.

## Data Availability

The data that support the findings of this study are available from the corresponding author upon reasonable request.
